# Composite Reliability of Workplace Based Assessment of International Medical Graduates

**DOI:** 10.15694/mep.2021.000104.1

**Published:** 2021-04-30

**Authors:** Balakrishnan Nair, Joyce M. W. Moonen – van Loon, Mulavana Parvathy, Cees P. M. van der Vleuten

**Affiliations:** 1 University of Newcastle; 2 Faculty of Health; 3 Centre for Medical Professional Development

**Keywords:** Composite reliability, reliability, programmatic assessment, International medical graduates, performance assessment, Workplace Based Assessment

## Abstract

This article was migrated. The article was marked as recommended.

Introduction

All developed countries depend on International Medical Graduates (IMGs) to complement their workforce. However, the assessment of their fitness to practice and acculturation into the new system can be challenging. To improve this, we introduced Workplace Based Assessment (WBA), using a programmatic philosophy. This paper reports the reliability of this new approach.

Method

Over the past 10 years, we have assessed over 250 IMGs, each cohort assessed over a 6-month period. We used Mini-Cex, Case Based Discussions (CBD) and Multi-Source Feedback (MSF) to assess them. We analysed the reliability of each tool and the composite reliability of 12 Mini-Cex, 5 CBDs and 12 MSF assessments in the tool kit.

Results

A reliability coefficient of 0.78 with a SEM of 0.19 was obtained for the sample of 236 IMGs. We found the MSF to be the most reliable tool. By adding one more MSF to the assessment on two occasions, we can reach a reliability of 0.8 and SEM of 0.18.

Conclusions

The current assessment methodology has acceptable reliability. By increasing the MSF, we can improve the reliability. The lessons from this study are generalisable to IMG assessment and other medical education programs.

## Introduction

International medical graduates (IMGs) make up to 30 % of the workforce in countries like Australia, United States, U.K. and Canada ( Patel *et al.,* 2018). All these countries have multicultural populations and hence their contribution to health care provision is culturally appropriate ( Pinsky, 2017). However their journey in the health care system is often challenging ( House of Representatives Standing Committee on Health and Ageing, 2012). The IMGs end up working in remote and unpopular locations and specialties. In spite of this, their scientific and academic contributions are significant. Approximately 18% of scientific publications are from IMGs and 18.3 % of professors are IMGs in the USA ( Khullar *et al.,* 2017). They give excellent care to their patients in spite of concerns from some quarters. In a study done on the outcome of 244,153 hospitalisations for congestive heart failure and acute myocardial infarction in Pennsylvania, treated by IMGs, there was no mortality difference compared to U.S. graduates ( Norcini *et al.,* 2010). In spite of this, the rates of disciplinary action against IMGs are higher than that for local graduates ( Alam *et al.,* 2017). There could be many reasons for this. Poor communication, lack of cultural awareness and issues with patient centred care are postulated as some reasons for this. Other causes could be economic pressures with resettlement in the new country, lack of orientation to the new health system and lack of mentorship, and performance assessments ( Hyder, 2017).

Because of the complexity of these issues, there had been suggestions to change the assessment for IMGs before they qualify to practice in the new country. For example, the International English Language Test System (IELTS) tests language proficiency and uses role players as one of the assessment tools. This may not be sufficient to test the linguistic skills of IMGs ( Tiffin *et al.,* 2014).

In recent years, Workplace Based Assessment (WBA) has become popular in medical education ( Norcini and Burch, 2007). The practice of medicine is a complex issue and medical knowledge alone will not be sufficient to practise.Oftenit is what the doctor *does* is more important than what the doctor knows for the individual patient and society. Because of this, there is more interest in performance based assessment in recent times ( Whelan *et al.,* 2002). Many undergraduate programs have introduced programmatic assessment using WBAs to assess the performance of the learner over a period of time. Programmatic assessment is when low stakes assessments are used in conjunction with immediate feedback leading to an aggregated summative decision making. This assessment will detect issues in the traditionally difficult areas to assess, like communication skills, teamwork and professionalism ( Wilkinson *et al.,* 2011). Most post graduate training programs are also introducing WBA with authentic assessment tools. The advantage of programmatic assessment is regular assessment from multiple assessors over a period of time with very frequent constructive feedback ( Chan and Sherbino, 2015). This will be assessment for learning rather than assessment of learning.

To remediate some of these issues, we developed a WBA program for IMGs working in our hospitals. The traditional pathway for IMGs in Australia is IELTS, followed by an MCQ examination and an OSCE examination ( Australian Medical Council, 2018). We offered a WBA program as an alternative and better option for IMG assessment. The Australian Medical Council accredited this program. We used Mini-Cex, Case Based Discussions (CBD) and Multisource feedback (MSF) as the main tools for assessment ( Nair *et al.,* 2012). This was a 6-month longitudinal performance assessment. We found this format was acceptable to the IMGs and assessors ( Nair *et al.,* 2015). Moreover, this assessment is cost effective and a good investment in the long term ( Nair *et al.,* 2014).

We have assessed over 250 IMGs over the past 10 years. While we know the reliability of individual assessment tools used in the WBA ( Castanelli *et al.,* 2019), we need to know the composite reliability of these tools when used in a tool kit ( Nair *et al.,* 2017). This paper is an extended analysis with a larger sample size. We believe that the lessons learnt can be used in other settings, both in undergraduate and post graduate assessments.

## Methods

IMGs who have passed the IELTS and MCQ examination have to wait to get into the OSCE clinical examination. Some of them are employed on a provisional registration to work as junior doctors in hospitals where there is doctor shortage. We set up a program in 2010 after getting accreditation from the AMC to evaluate their performance as an alternative to the 3-hour OSCE examination. The candidates attended a session where they were oriented and trained about the 3 assessment tools (Mini-Cex, CBD and MSF).

These assessment tools are well known and validated. The Mini-Cex was developed to test the clinical performance of the trainee. This is typically done in under 30 minutes, including time for immediate constructive feedback from the assessor ( Garibaldi *et al.,* 2002). The CBDs are to test the clinical reasoning and record keeping. The candidates select a patient whom they had looked after and the assessors will spend less than 30 minutes for assessment and feedback ( Norcini and Burch, 2007). Multisource feedback had been used in management for a long time and is becoming popular in performance-based assessment. The candidates nominate colleagues, both medical and nonmedical, and the assessors usually send in the evaluation. This had been reported to be a valid and reliable tool ( Miller and Archer, 2010).

Our assessment period was 6 months; the IMGs had to do 12 Mini-Cex assessments in medicine, surgery, women’s health, paediatrics, mental health and emergency medicine, 2 in each discipline. They were blueprinted to cover all domains including physical examination, history taking, counselling and prescribing. The candidates had to pass 8 cases, with at least one pass in each discipline, in order to pass the education program.

In the initial period, we used 7 CBDs and requested 12 MSF assessments on 2 occasions. We realised this was difficult to get and reduced the numbers to 5 CBDs and 6 MSF on 2 occasions. The IMG had to do 5 CBDs on patients they had managed to assess their record-keeping and clinical reasoning. They had to pass 4 out of 5 CBDs. Where possible, for the CBD and Mini-Cex assessments, we used different assessors.

At month one, they had to nominate 6 colleagues who had sufficient knowledge about their performance (3 medical and 3 nonmedical) for MSF. We had stipulated that the medical colleagues should be senior clinicians and the nonmedical colleagues should be nurses and allied health professionals. Once they nominated assessors, the rating forms were sent out from the central office to make the rating confidential and anonymous. The candidates were given the de-identified rating scores and a multidisciplinary team gave them constructive feedback. Remediation was offered to candidates if needed, including one to one communication skills training. At month 6, the candidates nominated another 6 different colleagues.

An executive committee, including clinicians and educators, oversaw the program and decided on pass /fail outcomes. If there were any procedural issues or appeal from the candidates, the assessment was reviewed by the Director. Only on less than 10 occasions were the candidates given a second chance for the assessment.

We had trained over 170 clinicians on WBA and assessment tools. They all attended a 3-hour calibration session before they were eligible to assess. At this session, they were given the rationale for this assessment and shown videos of the assessment scenarios. They independently marked each scenario, followed by feedback from experts in an interactive session. The emphasis on the training session was about multiple assessments by multiple assessors and immediate constructive feedback. The executive committee was able to review the assessments. We also did periodic feedback and upskilling sessions for them.

All assessments were scheduled by the administrator, with at least 2 weeks’ notice. All candidates knew the blueprint and the schedule of assessments. They attended a 3-hour orientation session before the program.

### Ethics

All candidates and assessors gave consent to evaluate the data. The research was approved by the Health Services Research and Ethics committee (approval number A.U.- 201607-03) of the Health Service.

## Results/Analysis

### Data analysis

We collected all completed MSF, Mini-Cex and CBD assessments. The Mini-Cex and CBD assessments contain 7 questions to be assessed on a 1-9-point scale. The MSF rating sheet contains 23 questions on a 1-5-point scale. The MSF assessment forms are different to medical and nonmedical colleagues, since different assessors are likely to see different behaviour of the candidates. Therefore, they are treated as separate measures of performance for the candidates and thus as different types of assessment. All these forms were validated in previous studies. To assure homogeneity among the assessments in the portfolio, we transformed the 5-point scale used in MSF to a 9-point scale (i.e. answer times 2 minus 1) in the dataset. In this transformation, each answer is multiplied by 2 and subtracted by 1, to assure that e.g. score “1”, “5”, “9” on the 9-point scale equals score “1”, “3”, “5” on the 5-point scale. For every assessment, the average score of all answered questions is determined and used in the calculations. Empty assessments were omitted from the analysis. Candidates for which the number of CBDs and/or Mini-Cex did not satisfy the quantitative requirements of 5 and 12, respectively, were excluded from the dataset, as well as the IMGs that did have less than 10 assessments for the MSF in total. The data of 236 IMGs are included in the dataset for analysis.

### Generalizability

For the generalizability study, which is completely performed in R ( R Core Team, 2019), we use a nested design where the unique assessment ( *i*) is nested in the facet of candidates ( *p*), *i:p.* The assessors are no facet in this design, as the set of assessors is very large and possibly unique per candidate because of the characteristics of the MSF. We determined the reliability and Standard Error of Measurement (SEM) for each of the three assessment types, using the required number of assessments as dictated by the program. And we performed a D-study for each assessment type with varying number of assessments per type.

Next, we analysed the composite reliability of the three types together using multivariate generalisability theory ( Brennan, 2001), combined in a portfolio, using a similar technique as described in Moonen - van Loon *et al.* (2013). Here, we also used the by the program required number of assessments as available in the dataset. To determine the composite universe score and composite error score that are needed for the reliability, the variances, covariances and absolute error scores of (combinations of) the available assessment types are combined using so-called weights, in which each type is assigned a percentage of impact on the composite reliability. By choosing a certain set of predetermined weights to apply in calculating the composite reliability, it gives assessors an indication on the importance of each assessment type in the complete set of available assessments while deciding of the performance of the candidates.

Finally, we changed the number of assessments to analyse the number of assessments per type that are needed to obtain a reliability ³ 0.80 and SEM £ 0.26, which are the widely used acceptable thresholds for reliability ( Crossley *et al.,* 2002), to see which changes in the assessment program could be made to reliably make high-stakes decisions on the performance of candidates.

### Results

The cleaned dataset consists of 7472 assessments of 236 candidates. Table 1 presents the statistics on the dataset, including the number of assessments of the 236 candidates available as well as the average and standard deviation on scores, and the required and average number of assessments per candidate.

**Table 1:** Descriptive statistics

	Case Based Discussion (CBD)	Multi Source Feedback (MSF)Medical colleague	Multi Source Feedback (MSF) Non-medical colleague	Mini-Cex
Number of assessments	1430	1596	1594	2852
Number of candidates	236	236	236	236
Average score (1-9-scale)	6.20	7.62	7.88	5.96
Standard deviation on the score (1-9-scale)	1.38	0.87	0.93	1.31
Number of assessments as required by the program	5.00	6.00	6.00	12.00
Average number of assessments	6.06	6.76	6.75	12.08

Performing the reliability analysis on each type of assessment using the required number of assessments, i.e. 5 Case Based Discussions (CBD), 6 Multisource Feedback (MSF) by medical colleagues and 6 by nonmedical colleagues and 12 Mini-Cex, we obtained the results presented in Table 2. The reliability coefficient is below 0.8 for each individual assessment type. We can conclude that the MSF is the most reliable tool in the portfolio. However, each type of assessment on its own will not lead to a reliability result with the currently used number of assessments.

**Table 2:** Reliability of each assessment type using the number of assessments as required by the program

	CBD	MSF Medical colleague	MSF Non-medical colleague	Mini-Cex
Reliability coefficient	0.40	0.61	0.59	0.52
Standard error of measurement (SEM)	0.58	0.32	0.34	0.36

**Figure 1:**
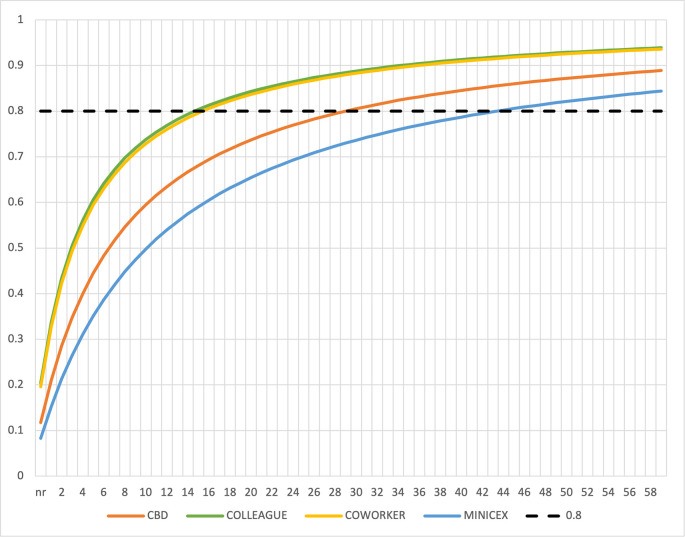
Reliability Coefficient (y-axis) for varying number (nr; x-axis) of Case Based Discussions (CBD), Mini-Cex (MINICEX), medical (COLLEAGUE) and nonmedical (COWORKER) Multisource Feedback (MSF) assessments

Figure 1 shows the reliability coefficient for varying numbers of assessments. To obtain reliable results for each assessment type individually, the program would have to do at least 30 CBD, 43 Mini-Cex and 16 MSF assessments per candidate over 2 rounds. For a 6-month program, this is not feasible for candidates and assessors.

However, in our program, results are taken together to contribute to the final pass/fail decision. Therefore, we calculated the *composite reliability* of the four types. The calculation of the composite reliability and SEM asks for a number of assessments per type and a weighting assigned to each type, where the sum of weights is equal to one, corresponding to the importance or impact of the assessment type in the complete assessment program. Similarly, as above, we use the, by the program, required number of assessments (5 CBD, 6 MSF per type and 12 Mini-Cex). We define the impact (or weight) per type to 13% CBD, 20% Mini-Cex and 67% MSF, equally split between the medical and nonmedical assessors. The assessments in the dataset following the requirements in the program lead to a composite reliability of 0.78 with a SEM of 0.19.

In the purported dataset, candidates select on average around 6.75 assessors of each type in the MSF. If we were to change the criteria on the number of assessors from 6 per type in total (3 in first month, 3 in 6 ^th^ month) to 7, then with the same weighting of the assessment tools, we obtain a reliable composite result of 0.80 with a SEM of 0.18.

## Discussion

Based on our data over the last 10 years, we believe, using 12 Mini-Cex, 5 CBD assessments combined with 6 MSF per round can provide an assessment program with satisfiable reliability for IMGs. As in any assessment program, the reliability should be balanced against acceptability, cost, educational impact and validity ( van der Vleuten and Schuwirth, 2005; Castanelli *et al.,* 2019). From our previous qualitative study, the acceptability is high from the learner perspective ( Nair *et al.,* 2015). They valued the immediate constructive feedback. The formative assessment program was an “educational journey” for them. They appreciated the opportunity to get to know the system and get acculturated. From the faculty point of view, they reported less pressure since this was a longitudinal assessment and they were part of a team of assessors. For the six-month program, the opportunity cost was 15,000 Australian dollars ( Nair *et al.,* 2015). This was acceptable to the health service and they saw this program as a long-term investment to produce safe and competent doctors, in areas where they were needed.

Any assessment on medical performance should be done using different tools, since practicing medicine is a complex activity and any single instrument will not fit the purpose. Hence any assessment should use multiple tools to provide breadth and to reduce the bias should use multiple observers.

To obtain reliable results for each assessment type individually, the program would have to include at least 30 CBD, 43 Mini-Cex and 16 MSF assessments from medical and non-medical colleagues. For a 6-month program, this is not feasible for candidates and assessors. However, when these assessments are combined, we can get a reliable assessment with 31 assessments in total. Moreover, when the assessment is spread out over 6 months using different assessors, the assessment fatigue is minimised and make it more acceptable to the busy faculty. In fact, we see that candidates collected more MSF assessments than required on average, for both medical and nonmedical colleagues, indicating that a very small increase in the required number of assessments seems feasible, leading to a reliability of 0.8.

The MSF was the most reliable tool in our study. It is not surprising since this is based on a more longitudinal observation of the trainee. This is consistent with the previous studies ( Miller and Archer, 2010; Castanelli *et al.,* 2019).

Another strength of our study is its validity. As the trainees themselves described, this is an assessment done on real patients, by real clinicians in real hospitals and is an educational journey. They appreciated the immediate feedback and the supervisors reported progress of the candidates over the 6 months ( Nair *et al.,* 2015). So we believe our program fulfils all the requirements of a good assessment program, including reliability, acceptability, cost and educational impact ( van der Vleuten and Schuwirth, 2005).

However, this program is done by one centre and the other centres may have a different experience. As in any new program, faculty buy-in and training was challenging. It will be good to study the reliability and acceptability in other sites doing similar programs. However, we think what we have learned can be adapted in different educational settings including undergraduate and postgraduate program and should not be confined to IMG assessments.

## Conclusion

We believe the WBA program has good composite reliabilty and by adding 2 extra MSF assessments, we can increase the reliability to 0.8. The lessons learned can be extrapolated into other assessments, both in undergraduate and postgraduate medical assesments. This program is acceptable to the learners and assessors and is cost effective.

## Take Home Messages

• Performance assessment is more important than competency assessment
• A programmatic assesment with different tools and multiple assessors will give good reliability
• The assessments should be blue -printed
• Constructive feedback in education is the key to improving performace

## Notes On Contributors

Professor Balakrishnan Kichu Nair, MD, FRACP, FRCP is the Professsor of Medicine and Associate Dean at the Medical School in Newcastle, Australia. He is the Director of Continuing Medical Professional Development Unit at Hunter New England Health and is the Director of Educational Evaluation at Health Education and Training Institue of NSW. ORCID: https://orcid.org/0000-0002-9100-4298

Dr Joyce M. W. Moonen - van Loon, PhD, is from the department of Educational Development and Research at Maastrict University. She is assistant professor, member of the taskforce “Instructional design and E-learning” with a focus on the use and implementation of portfolios. She has a background in Econometrics and received a PhD in Operations Research from Maastricht University in 2009. ORCID: https://orcid.org/0000-0002-8883-8822

Dr Mulavana Parvathy, MBBS, FRCGP is the Director of the IMG Program at the Hunter New England Health at Newcastle and is a family physician.

Professor Cees van der Vleuten, PhD, has been at the Maastricht University in The Netherlands since 1982. In 1996 he was appointed Professor of Education and chair of the Department of Educational Development and Research in the Faculty of Health, Medicine and Life Sciences (until 2014). Since 2005 he has been the Scientific Director of the School of Health Professions Education (until 2020). He mentors many researchers in medical education and has supervised more than 90 doctoral graduate students. His primary expertise lies in evaluation and assessment. ORCID: https://orcid.org/0000-0001-6802-3119
