# Supplementary material for: Composite Reliability of Workplace Based Assessment of International Medical Graduates
Source: MedEdPublish (2016). 2021 Apr 30;10:104. [Version 1] doi: 10.15694/mep.2021.000104.1 (PMC10939524; doi:10.15694/mep.2021.000104.1)
Supplement: Peer review history

# Reviewer report: Reviewer response for version 1

**DOI:** 10.21956/mep.19097.r27528

**Published:** 2021-05-10

**Authors:** Lorna Davin

**Affiliations:** 1 University of Notre Dame, Australia

This review has been migrated. The reviewer awarded 5 stars out of 5 It is very interesting to read the nuanced outcomes of this paper addressing an important area of medical education, our diverse medical workforce, and patient care. I especially appreciate the take-home message which emphasizes the importance of performance assessment over competency assessment. The approach taken allows the International Medical Graduates (IMGs) to be assessed within the messiness of their practice recognizing and embracing the social and cultural complexities of patient-care – which adds a depth of authenticity. The acceptance and buy-in of faculty is also significant. It is interesting to note how the longitudinal nature of the program, and the shared workload, reduced individual pressures of faculty.The use of Multisource feedback, across the disciplines, with an emphasis on support and remediation, over time, is an obvious strength of this well-considered and managed program. The research outcomes emphasize validity and reliability. However, the program also adds a layer of transparency to the IMG assessment process, and in doing so I would argue provides the learner with a fairer and more equitable assessment, supporting this specific group of learners, and much needed part of our medical workforce, while they transition to clinical practice in an unfamiliar place and culture.The authors are to be congratulated on this most useful contribution which adds to our understanding of how to better support learners and faculty while indirectly enhancing future patient care.

Reviewer Expertise:

NA

No decision status is available

# Reviewer report: Reviewer response for version 1

**DOI:** 10.21956/mep.19097.r27525

**Published:** 2021-05-07

**Authors:** Karen D'Souza

**Affiliations:** 1 Deakin University

This review has been migrated. The reviewer awarded 5 stars out of 5 The authors of this paper are leaders in the field of WBA and programmatic assessment. I was impressed by this group's work as they aimed to conduct the fairest, most reliable program of clinical assessment for IMGs whilst also considering the feasibility of delivering such a program of in the clinical setting. I applaud the group for their demonstration of the statistical analysis supporting their conclusions. Of particular note is the assessment performance of the MSF method of collecting datapoints, not only from medical supervisors but also other health professionals to build the most comprehensive and holistic clinical performance review. Their program of WBA also builds in feedback at ideal time points within the 6 month period of study - i.e. one MSF early, and one towards the end of 6 months, thus providing the opportunity for early feedback to shape the training of the IMGs, and then a later checkpoint for the program directors and trainees to review whether their training goals have been achieved to a sufficient degree. Sharing the rich feedback gained from the MSF forms with the IMGs allows the full potential of these assessments to be realised. Their results should be highly generalisable to other training contexts beyond IMG assessment.

Reviewer Expertise:

NA

No decision status is available

# Reviewer report: Reviewer response for version 1

**DOI:** 10.21956/mep.19097.r27523

**Published:** 2021-05-06

**Authors:** Dujeepa D. Samarasekera

**Affiliations:** 1 National University of Singapore

This review has been migrated. The reviewer awarded 4 stars out of 5 Interesting paper and enjoyed reading it. The paper addresses an important challenging area in medical education and evaluating the competencies of physicians who migrate from one practice setting to another. The authors have discussed a more wholistic evaluation of an IMG's competence and their actual practice performance. Another strong point in this study is that they have collected data from over 200 IMGs in a 10-year period to make their final conclusions. Another important highlight is how they have designed this process aligned to the principles of programmatic assessment incorporating day-to-day workplace-based assessments in a practical and useful way both to the IMG and the supervisors. I am certain that this paper will assist many of us who are planning clinical assessments to evaluate trainees' performance for safe and effective practice. Apart from some minor limitations highlighted by other reviewers above, I am happy with the work done.

Reviewer Expertise:

NA

No decision status is available

# Reviewer report: Reviewer response for version 1

**DOI:** 10.21956/mep.19097.r27527

**Published:** 2021-05-03

**Authors:** Chris Roberts

**Affiliations:** 1 OPME - University of Sydney NSW AUSTRALIA

This review has been migrated. The reviewer awarded 4 stars out of 5 Work based assessment is an important area of medical education research and policy development. Many MedEdPublish readers who are clinicians will either have experienced or be expected to assess student/trainee in the work place. This paper evaluates the reliability of Workplace Based Assessment (WBA) of International Medical Graduates (IMGs) in particular establishing the composite reliability of a WBA toolset consisting of MiniCEX CBD, and MSF. The authors have available 10 years of data, (7472 assessments of 236 candidates) and gives a good account of providing further validity evidence on a long standing system outside some of the main centres in the US and UK. It assures the stability of the estimates of the reliability/standard error of measurement of the tools used alone and in combination in this context. The paper also has value in describing a programmatic approach to standard setting and decision making at the end of the 6 month assessment period on the composite reliability of multiple samples of behaviour using multiple judges. The deepening of the validity evidence is important for many institutions internationally who offer WBAs but are keen to demonstrate robust assessment of learning for their major stakeholders in this kind of assessment. The gold standard of > 0.8 perhaps drives us towards homogeneity in the various assessment types? Would reducing this the more realistic r= 0.7 help in this regard to have a more heterogenous sample of behaviours for the composite? I was interested to know for future research how the qualitative feedback received by trainees might enrich the progression decisions of the executive committee that considers the program of WBAs.

Reviewer Expertise:

NA

No decision status is available

# Reviewer report: Reviewer response for version 1

**DOI:** 10.21956/mep.19097.r27524

**Published:** 2021-05-02

**Authors:** Tarun Sen Gupta

**Affiliations:** 1 James Cook University

This review has been migrated. The reviewer awarded 5 stars out of 5 I enjoyed reading this paper from some of the leading proponents of work based assessment. Kichu Nair’s team have led the research agenda in WBA, underpinning the strong educational and practical aspects with a sound and detailed evaluation. Their data set is substantial - 250 trainees over 10 years - and the results likely to be significant in many other jurisdictions, as the authors note.The programmatic aspect of this work is particularly important, presenting a ‘composite reliability’ in addition to reporting each component, which adds further weight to the argument regarding combining multiple assessment formats. Furthermore, in the time of COVID, educators world-wide are looking at alternatives to ‘big bang’ events, so this approach presents a credible option.I also appreciated the positive acknowledgement of the important work that IMGs do, and their very significant contribution to medical workforce in many settings. There is also important local and international context and sufficient detail to allow others to replicate this program in their own environment.The SEM quote of 0.18-0.19 did look very low to me, but I note this is on a reduced scale (9 points, reduced in some analyses to 5), not a percentage. This is a detail the authors may wish to clarify in any update. The wording ‘by the program required...’ in reference to the number of assessments is also a little clumsy.These are only minor quibbles in what is an important addition to the literature. The authors’ comments on the number of assessments required for desired reliability (Figure 1) and the observation about the utility of MSF are worth noting as important findings in themselves. I also note their description of training clinic as in the various techniques before they are ‘eligible to assess’ which is often omitted or done poorly.I think this article provides some useful evidence on which to further develop our assessment programs in a post-COVID world.

Reviewer Expertise:

NA

No decision status is available

# Reviewer report: Reviewer response for version 1

**DOI:** 10.21956/mep.19097.r27522

**Published:** 2021-05-01

**Authors:** Sankar Sinha

This review has been migrated. The reviewer awarded 5 stars out of 5 Prof Nair and his co-authors of this article are renowned International experts on developing the Workplace-Based Assesment program for the International Medical Graduates. This article is the culmination of their ten-year experience on Mini-Cex, Case Based Discussions (CBD) and Multisource feedback (MSF) assessment tools. It was indeed a pleasure to read this well-written concise article demonstrating the value of composite reliability in the WBA program. It is heartening to read their concluding statement “The lessons learned can be extrapolated into other assessments, both in undergraduate and postgraduate medical assesments.” I have been trying to implement this strategy for the Interns at our hospital based on the evidence from Prof Nair’s experience of WBA for the IMGs.

Reviewer Expertise:

NA

No decision status is available

# Reviewer report: Reviewer response for version 1

**DOI:** 10.21956/mep.19097.r27526

**Published:** 2021-05-01

**Authors:** Ian Wilson

**Affiliations:** 1 International Medical University

This review has been migrated. The reviewer awarded 4 stars out of 5 This paper describes a response to a difficult problem. The assessment of international Medical graduates (IMGs) has been problematic for many years. Th use of WBA in a composite way in this paper demonstrates an effective, reliable method. The composite outcomes ensure a reliable method of assessment that is acceptable to those being assessed and produces results that enable rational decisions to be made.Th authors of this study have demonstrated a method of assessing IMGs that will produce results that will lead to a much more reliable an effective method of ensuring the competence of those doctors about to enter practice.

Reviewer Expertise:

NA

No decision status is available
